# An exploratory mixed-methods assessment of private health sector readiness for reporting through the Ministry of Health and Child Care Health Information System in Zimbabwe

**DOI:** 10.1371/journal.pdig.0001566

**Published:** 2026-07-21

**Authors:** Memory Chimsimbe, Tariro C. Mando, Munyaradzi Murwira, Juliet G. Nyamasve, Eliud Wekesa, Anne Njeri, Patron Mafaune, Jennifer Harris Requejo, Manes Munyanyi, Winston Chirombe, Simukai T. Zizhou, Oona Campell, Richard Makurumidze

**Affiliations:** 1 Department of Global, Public Health and Family Medicine, Faculty of Medicine and Health Sciences, University of Zimbabwe, Harare, Zimbabwe; 2 Department of Rehabilitation Sciences, Faculty of Medicine and Health Sciences, University of Zimbabwe, Harare, Zimbabwe; 3 Health Services, Kwekwe City, Midlands Province, Zimbabwe; 4 Africa Population Health and Research Centre, Nairobi, Kenya; 5 Global Financing Facility for Women, Children, and Adolescents, World Bank Group, New York, United States of America; 6 Ministry of Health and Child Care, Harare, Zimbabwe; 7 London School of Tropical Medicine and Hygiene, London, United Kingdom; Yonsei University College of Medicine, KOREA, REPUBLIC OF

## Abstract

Zimbabwe’s healthcare system is a blend of public and private sectors, yet a substantial gap remains in integrating private-sector data into the Ministry of Health and Child Care’s (MoHCC) Health Information System (HIS) to achieve a comprehensive national picture. This exploratory descriptive cross-sectional study used a mixed methods approach to assess the organisational readiness of Zimbabwe’s private healthcare sector for reporting through MoHCC HIS. The key facilitators and barriers influencing the successful integration of private healthcare data into MoHCC HIS. Data were collected through an online survey (n = 49), and an interview guide was used to conduct in-depth interviews (n = 24) with private health facilities stakeholders using constructs such as structural readiness, technological readiness, resource availability, and skill and knowledge readiness. Informed written consent was obtained from the participants for the in-depth interviews. Ethical approval was granted by the Joint Research Ethics Committee JREC/438/2024. Epi Info 7 was used for calculating frequencies and proportions for the quantitative data. Thematic analysis was done for the qualitative data. The private sector was partially ready to report through the MoHCC HIS, as evidenced by facilities’ willingness and leadership commitment to report, the availability of infrastructure, and the presence of data management systems. The study highlights potential facilitators such as enhancing technological readiness, improving staff training, and fostering stronger communication between the public and private sectors. Key challenges identified include inadequate legal frameworks supporting mandatory reporting and limited resources within private facilities. This assessment provides vital information for integrating private sector health data into Zimbabwe’s national Health Information System. Addressing existing gaps and leveraging identified facilitators, stakeholders can enhance the quality of health data available for policy planning and resource allocation, ultimately improving health outcomes.

## Author summary

Zimbabwe’s healthcare system comprises both public and private providers; however, there is currently insufficient sharing of health data between private facilities and the Ministry of Health and Child Care. This lack of data exchange hinders a comprehensive national understanding of health trends and requirements. To investigate this issue, we conducted a study to assess the preparedness of private health facilities to contribute their data to the national Health Information System. The research utilised a combination of surveys and interviews involving 73 representatives from private health facilities.

The findings indicate that private facilities are partly ready to share their data. Many possess the necessary technology and are eager to engage, provided they receive adequate support from leadership. Nonetheless, several challenges persist, including the absence of legal mandates for reporting and deficiencies in staff training. The study further identified constructive measures to advance, such as enhancing communication between the public and private sectors, strengthening staff capacity, and reinforcing technical systems.

This evaluation provides insightful information for the government and health stakeholders. By tackling the challenges highlighted and leveraging the strengths observed in the private sector, Zimbabwe can progress toward a more comprehensive and reliable health data system that will facilitate improved planning, decision-making, and health services for everyone.

## Introduction

### The Zimbabwean public and private healthcare structures

Zimbabwe’s healthcare framework consists of both public and private providers. Each has its own management, funding, and working methods.

The private healthcare sector is generally understood as all organisations and individuals providing health services that are not owned or directly managed by governmental bodies [1]. It includes a wide variety of participants, from profit-driven entities—such as private hospitals, clinics, manufacturing facilities, and individual healthcare providers—to non-profit organisations, like mission hospitals and Non-Governmental Organisations (NGOs). In numerous low- and middle-income countries (LMICs), the private sector plays a vital, albeit often fragmented, role within the healthcare system. It supplies additional resources, improves service availability, and usually offers specialised or advanced care that may not be accessible in the public sector [2]. The private sector mainly operates on market forces and relies on out-of-pocket payments, private insurance, and, sometimes, donor funding. There are no standard or mandatory protocols for sharing health information with national authorities.

The public sector is governed by the Ministry of Health and Child Care (MoHCC). It includes services provided by local government and other ministries, such as Defence and Education, Home Affairs, and Prisons, offering health services in both rural and urban settings. Public institutions operate within a centralised framework with set procedures, and they must follow a national reporting system.

On the other hand, the private sector operates independently, ranging from small, solo-practitioner clinics to large, well-equipped hospitals. The Health Professions Authority (HPA) reports that there are approximately 5,377 registered health facilities in Zimbabwe, including both sectors [3]. However, the operational Health Information System (HIS) managed by the MoHCC actively monitors only about 1,848 facilities, highlighting a significant gap in the formal registration and data capture of a considerable number of private providers [4]. This structural distinction leads to fundamental differences in operations, funding, and accountability. Public facilities are part of a centralised reporting structure, while private facilities operate under market forces and lack a standardised requirement to share data with national authorities. The Zimbabwe Demographic Health Survey (ZDHS) 2023–24 highlight the considerable role of the private sector in service delivery; for example, approximately 30–35% of family planning clients [5] and 6.3% of deliveries take place in private or mission facilities [6]. This substantial market presence renders the omission of private sector data from national health statistics a notable weakness, obscuring a complete understanding of the nation’s health status, disease burden, and resource requirements.

### Health information systems and current limitations in Zimbabwe

An effective national HIS is a critical element of a robust health system, vital for planning, management, and evidence-based decision-making [2]. In Zimbabwe, the MoHCC’s Department of Health Information and Data Analytics oversees the national HIS, utilising the District Health Information System 2 (DHIS2) software for data aggregation, analysis, and storage. This unit is responsible for clearly defining key health indicators, establishing appropriate data collection methods and procedures, and ensuring that well-trained and motivated health information personnel are in place. Data are collected from all levels of the healthcare system, including primary, secondary, tertiary, and quaternary care in both public and private sectors. The system gathers information through a series of paper-based tools, such as hard-copy registers and tally sheets, that operate from the primary health facility to the district, for onward transmission to the provincial and national levels [7].

Despite advancements in modernising the HIS through initiatives such as the iMpilo Electronic Health Record and the electronic Logistics Management Information System, a significant weakness remains the inadequate integration of data from the private sector. The current mechanism largely relies on voluntary reporting or on specific conditional arrangements, such as the Expanded Programme on Immunisation vaccine provision. Zimbabwe has established multiple public health surveillance systems to monitor disease outbreaks, follow health trends, and inform public health interventions. These systems detect infectious diseases, such as cholera, tuberculosis, and HIV, and non-communicable diseases. However, the Public Health Act does not mandate comprehensive data reporting from private facilities, except in cases of outbreak-prone diseases [8]. As a result, much private health data covering diseases, conditions, and inpatient statistics remains outside the national system, leading to a glaring data gap that hampers the comprehensiveness, accuracy, and utility of national health

### Contribution of this paper

Successful integration of private-sector data into a national HIS depends critically on the initial readiness, preparedness, and capacity of the private facilities themselves. While the data gap is recognised, there is a dearth of empirical evidence in Zimbabwe [8]. Zimbabwe on the operational readiness of private health facilities to participate in the national HIS. Previous discussions have primarily focused on policy gaps or technical system design without grounding them in the on-the-ground realities, constraints, and perceptions of private-sector providers [5].

This paper provides a critical, evidence-based assessment to address this knowledge gap. Using a mixed-methods approach guided by the Organisational Readiness for Change framework [9]. The study was guided by the Organisational Readiness for Change (ORC) framework [10], which conceptualises readiness as both the collective determination to implement change (change commitment) and shared confidence in the ability to do so (change efficacy). This framework was selected because it encompasses both psychological and structural dimensions of organisational change—essential for assessing private sector readiness for HIS integration, which requires not only technical infrastructure but also provider willingness to adopt new reporting practices.

The ORC framework directly shaped each research objective through its core constructs. Structural readiness informed Objective 1 on the facilities’ infrastructure for reporting. Resource availability shaped Objective 2 on staff skills and resources. Psychological readiness underpinned Objective 3, which focused on provider perceptions of integration. Leadership readiness guided Objective 4 on leadership’s role. The framework’s emphasis on identifying facilitators and barriers directly shaped Objective 5. These constructs were operationalised in both quantitative and qualitative tools to ensure theoretical consistency.

We move beyond theoretical integration challenges to empirically evaluate the structural, technological, resource-based, and attitudinal readiness of Zimbabwe’s private healthcare sector. Our assessment specifically sought to:

1. Identify private facilities’ structural and technological readiness for reporting through the MoHCC HIS, 2024
2. Determine the resources, staff skills and knowledge readiness for for reporting through the MoHCC HIS, 2024
3. Evaluate healthcare providers’ perceptions towards integrating with the MoHCC HIS, 2024.
4. Determine leadership readiness for reporting through the MoHCC HIS, 2024
5. Identify key facilitators and barriers for successful integration of the private sector into the MoHCC HIS, 2024.

By doing so, this study offers a nuanced, ground-level perspective that is essential for formulating feasible, context-appropriate strategies. The findings aim to inform policymakers, the MoHCC, and development partners on the specific investments, policy adjustments, and support mechanisms required to transition from a fragmented to an inclusive, comprehensive national health information landscape. Ultimately, bridging this data divide is a prerequisite for achieving accurate health intelligence, equitable resource allocation, and improved health outcomes for Zimbabwe’s entire population.

## Materials and methods

### Ethics statement

Permission to conduct the study was obtained from the MoHCC. Ethical approval was obtained from the Joint Research Ethics Committee JREC/438/2024. The purpose of the study, expected outcomes, process, and confidentiality of responses were explained to all potential respondents, who were invited to participate. Written informed consent was obtained from the study participants for the in-depth interviews. To ensure confidentiality, data collection tools were anonymised.

### Study design

We conducted an exploratory descriptive cross-sectional study using a concurrent mixed-methods approach [11], integrating quantitative and qualitative interviews equally, to thoroughly examine the preparedness of Zimbabwe’s private health institutions to submit reports via the MoHCC HIS. The mixed-methods approach allowed for a comprehensive assessment of readiness levels while providing detailed insights into systemic, operational, and attitudinal factors that influence data integration. As a result, it offered a robust, triangulated evidence base to inform practical policy and practice recommendations aimed at enhancing the private sector’s participation in national health reporting.

### Study setting

Private healthcare providers in Zimbabwe’s ten provinces participated in an online national survey for the quantitative cross-sectional study. The qualitative component of the study was conducted in seven private health institutions in Harare Metropolitan Province (Chitungwiza and Harare), all registered under the Private Healthcare Association of Zimbabwe (PHAZ).

### Study participants

#### Quantitative Component.

The study participants were interdisciplinary health professionals, including pharmacists, medical doctors, dentists, radiologists, laboratory scientists, nurses, occupational therapists, and physiotherapists. They fulfilled these criteria: registered with their respective professional organisations in Zimbabwe; employed at or owning a private healthcare facility or individual private practice, and engaged in or accountable for health data management or reporting at their establishment.

#### Qualitative component.

The study unit was a selected private health institution from Chitungwiza and Harare. Facilities qualified if they were: privately owned and registered with the Private Healthcare Association of Zimbabwe (PHAZ), and not consistently submitting data to the MoHCC HIS, specifically to evaluate readiness among entities that do not report. Within the selected facilities, key informants were deliberately selected for their positions and knowledge, including managers/proprietors, nurse managers, administrators, Information and Communication Technology (ICT) officers, and businesspersons.

### Sample size determination and sampling

#### Quantitative Component.

The calculated sample size using the Dobson formula, za2 x p (1-p)/ delta2, assuming unknown p = 0.5 for maximum sample size with a non-response rate of 10%, was 423.

The study employed purposive sampling of members of professional associations in Zimbabwe namely Health Professions Association, PHAZ, Zimbabwe Medical Association (ZIMA), Zimbabwe Women Doctors Association (ZWDA), Zimbabwe Physiotherapy Association (ZPA), Zimbabwe Association of Occupational Therapists (ZAOT), Zimbabwe Nurses Association (ZINA), College of Primary Health Care Physicians of Zimbabwe (CPCPZ), Zimbabwe Dental Association, Pharmacist Council of Zimbabwe, Medical Laboratory and Clinical Scientists Council Of Zimbabwe, Zimbabwe Association of Family Therapists and Professional Counsellors (ZAFTPC) who are in private practice target professionals with direct experience in private sector health information management and invited them to participate in the quantitative survey and 49 participants responded each from different facilities.

#### Qualitative component.

The Private Healthcare Association of Zimbabwe list was used as the sampling frame. Simple random sampling was used to select 20 private hospitals from the Private Healthcare Association of Zimbabwe list, which has 45 facilities. A total of 20 facilities were initially selected from this list using simple random sampling (random number generator) to ensure an unbiased starting group. Facilities were sampled from Chitungwiza and Harare. Sampling was based on the size of the facilities and the availability of various services offered. Private hospitals providing inpatient or outpatient services, or maternal and neonatal services, laboratories, pharmacies, radiology services, and rehabilitation services were selected from this list of 20. The study primarily focused on those who did not report and assessed their readiness to report. The research team reached out to the managers of the 20 chosen facilities by phone to present the study, confirm eligibility, and invite them to participate. Recruitment progressed methodically until seven facilities agreed to participate; this number was determined based on thematic saturation and constrained by the study’s budget and timeline.

The resulting sample encompassed a variety of facility types (such as hospitals and clinics) that provided diverse services (inpatient/outpatient care, maternal health, laboratory, pharmacy). After securing the agreement from the facilities, the research team worked with the facility manager to intentionally select 2–4 key informants from each site whose roles involved oversight of data, administration, or technology. Potential participants were approached in person, given a comprehensive information sheet, and invited to participate in a face-to-face interview.

### Data collection methods and tools

#### Quantitative Component.

An online data collection survey was created in Google Forms based on the constructs of the organisational readiness for change framework. Its design was theoretically based on the Organisational Readiness for Change (ORC) framework developed by Holt et al, which is a validated model commonly used to evaluate how prepared organisations are for systemic change. This framework suggests that readiness involves both the collective determination of organisational members to implement a change (change commitment) and their shared confidence in their ability to achieve it (change efficacy) [5].

### Instrument development

The survey was designed to translate key concepts from the ORC framework that are pertinent to HIS integration:

The questions were used to collect data on organisational structures and ICT infrastructure that support HIS reporting, data collection tools used in the private sector, beliefs and perceptions of healthcare providers towards HIS reporting, the role of leadership in private healthcare facilities, and barriers and facilitators influencing the successful integration of private healthcare data into the MoHCC HIS*. Respondents were prompted to consider specific elements such as workflow inefficiencies, organisational culture, existing incentives, and technical compatibility challenges. By providing short examples, the aim was to elicit richer and more actionable data. After defining these items, the following phases were to validate and refine the questionnaire to ensure its quality.*

To ensure content validity, a panel of five experts in health informatics, public health, and health systems research reviewed the draft questionnaire. The tool was then pretested with five health professionals from private facilities not included in the main study.. This thorough evaluation helped refine the questions, improve conformity to HIS reporting, and remove any repeated items. Their feedback helped refine the questions, enhance compliance with HIS reporting requirements, and remove any duplicate items.

The research team reached out to the leaders of each association to explain. A standardised invitation message, which included a brief overview of the study, eligibility requirements, and a link to the confidential online survey (hosted on Google Forms), was sent out by the associations’ administrators, along with a request for their permission to engage with their members.

The survey link was shared through two main channels that are well-regarded within the professional community: professional WhatsApp groups, where administrators shared the invitation and link in their official association groups. Official email lists: the invitation was distributed to members using the association’s bulk email systems. These channels were chosen because they are trusted entities within the healthcare sector, guaranteeing the access and trustworthiness needed to gather high-quality responses from relevant stakeholders. The final online survey was shared with professional associations, namely HPA, PHAZ, ZIMA, ZWDA, ZPA, ZAOT, ZINA, CPCPZ, the Zimbabwe Dental Association, the Pharmacist Council of Zimbabwe, the Medical Laboratory and Clinical Scientists Council of Zimbabwe, and ZAFTPC. The online survey made it possible to reach many participants nationwide and to observe the reactions being collected in real-time. To enhance response rates, reminder messages were sent weekly through the same channels over a four-week data collection timeframe (March - April 2024). Participation was entirely voluntary and anonymous. Implied consent was acquired when participants agreed to complete and submit the online survey.

#### Qualitative component.

The qualitative data were collected using a semi-structured interview guide, which was also based on the ORC framework to maintain theoretical consistency with the quantitative data [5]. A meeting was arranged at a time and in a private location within the facility to ensure privacy and minimise interruptions. Before each interview, the researcher obtained written informed consent by clearly outlining the study’s purpose, procedures, potential risks and benefits, and measures to ensure confidentiality. With consent, the interviews were audio-recorded. Participants were assured that their identities and facility identifiers would remain anonymous in all transcripts and reports. Qualitative data were collected through face-to-face interviews with key informants in seven private health institutions in Harare and Chitungwiza. Participants were purposively selected based on their roles in health information management and availability on the day of data collection. Each session lasted between 30 and 60 minutes and was guided by a structured interview tool explicitly developed for private healthcare providers. The tool covered domains such as organisational structures, technological capacity, resource allocation, staff skills, leadership commitment, and external influences on reporting to the MoHCC HIS. Before full implementation, the guide was pretested at one private facility in Harare to refine question wording and flow; insights from the pilot were incorporated into the final instrument. Written informed consent was obtained from all participants, and with permission, interviews were audio‑recorded and supplemented by detailed field notes. Interviews were conducted in English or Shona, depending on participant preference, and all recordings were transcribed and anonymised to protect confidentiality. Data collection continued until thematic saturation was reached across the facilities.

The simultaneous application of the survey (which measures readiness levels) and the interview guide (which qualitatively interprets them) enabled methodological triangulation. This strategy enhanced the overall validity of the results by aligning or elaborating on the fundamental concepts of organisational readiness in the private health sector in Zimbabwe.

### Patient and public involvement

No patients were involved in the design of this study, which focused on health facilities and health professionals. The public was involved in the dissemination of the findings, which will motivate participation and improve reporting of private-sector facilities to the MoHCC HIS.

### Data analysis

#### Quantitative Component.

The quantitative data were analysed using descriptive statistics. Initially, a Microsoft Excel worksheet with survey responses was downloaded from Google Forms. Following that, the data were verified for completeness and cleaned. The cleaned data were then uploaded to Epi Info 7, version 7.2.6.0, a statistical software tool for analysing public health data. This software made it easier to calculate frequencies and percentages, providing a more comprehensive view of the collected numerical data. The results were presented systematically in tables.

#### Qualitative component.

The qualitative data underwent thematic analysis, utilising a mixed approach that combined deductive codes drawn from the Organisational Readiness for Change framework with inductive codes generated directly from the interview transcripts. To ensure accuracy and rigour, two authors independently coded all transcripts. A third team member was responsible for checking the codebook and verifying the coding outputs. Coding was systematically managed using Microsoft Excel. Any coding conflicts were resolved through consensus meetings, with a senior team member arbitrating any persistent disagreements. The research team cooperatively finalised the resulting themes. Throughout this process, reflexive diaries were maintained to document analytical decisions, thereby enhancing the transparency of the analysis.

The research utilised a concurrent triangulation approach, combining quantitative and qualitative data at the interpretation phase to gain a thorough understanding of private-sector preparedness. Quantitative survey results revealed the levels and trends of preparedness across significant areas (such as technology and skills). At the same time, qualitative interview insights provided context, viewpoints, and the fundamental reasons behind those trends. The findings were integrated during analysis, enabling statistical trends to be enhanced and clarified by narrative insights, thus strengthening the validity and richness of conclusions through methodological triangulation

## Results

### Characteristics of Zimbabwe’s private healthcare facilities and professionals

#### Quantitative Component.

A total of 49 health facilities participated in the quantitative assessment. Pharmacies constituted the most significant proportion of facilities, at 18 (37%), followed by surgery centres, at 11 (23%), and medical centres, at 6 (12%). Rehabilitation clinics accounted for 4 (8%), while dental surgeries comprised 3 (6%). The other seven facility types each represented 1 (2%).

Regarding the respondents’ profession, pharmacists were the majority, accounting for 20 (41%), followed by medical doctors at 18 (37%). Physiotherapists and dentists each made up 3 (6%), while laboratory scientists and occupational therapists each accounted for 2 (4%). Nurses were the smallest group, contributing 1 respondent (2%), as shown in Table 1.

**Table 1:** Characteristics of Zimbabwe Private Healthcare Professionals, 2024.

Variable	Frequency n = 49 (%)
**Profession of the respondents**	
Pharmacist	20 (41)
Medical Doctor	18 (2)
Physiotherapist	3 (2)
Dentist	3 (2)
Laboratory scientist	2 (2)
Occupational therapist	2 (2)
Nurse	1 (2)
The facility has been in operation for	
Less than 5 years.	19 (38)
5-10 years	13 (27)
More than 10 years	17 (35)

#### Qualitative Component.

Twenty-four participants from the seven selected facilities were included in the qualitative component (Table 2).

**Table 2:** Characteristics of Zimbabwe Private Healthcare Professionals, 2024.

Variable	Frequency n = 24
Nurse Managers	6
Nurses	9
Proprietor	1
ICT Managers	5
Administrators	3
Total	24

### Structural and technological readiness of Zimbabwe’s private healthcare facilities for reporting through the MoHCC HIS, 2024

Among the five designated Health Information Officers (HIOs), three possessed diplomas in Health Information and Medical Records Management. One officer held both a certificate in Health Information and Medical Records Management and a bachelor’s degree in information technology. In contrast, the remaining officer held a bachelor’s degree in computer science.

The assessment showed that most facilities relied on paper-based health information systems (followed by hybrid systems and those without any system 10 (20%)). Electronic health records (9, 19%) and electronic medical records (4, 8%) were less common. Regarding health information software, Health 263 was the most widely used, at 27 (55%), while other tools, such as Dispense Ware 6 (12%) and Medi-Plus 2 (4%), had limited adoption. A notable 11 (23%) facilities reported not using any health information software.

Private healthcare facilities used both paper-based and electronic systems, showing a diversity of data management strategies. One key informant remarked the following:

“We are currently using paper-based forms for reporting to the City Health Department, which supplies us with vaccines for the under-fives, and we mostly use the monthly Tally (T5) forms. For the electronic system, we use TriMed for patient management, billing, and the generation of financial reports” (Nurse Manager 3).

The International Classification of Diseases, Tenth Revision, Clinical Modification (ICD-10) was used; no additional formal standards were noted. The respondent expressed herself as follows: “We use ICD-10 to code diseases and medical conditions data, and it is the only standard that we are currently using at this hospital.” (ICT Person 2) as shown in Table 3.

**Table 3:** Current health information software in use in Zimbabwe’s private healthcare facilities, 2024.

Variable	Frequency n = 49 (%)
Health Information software currently in use	
Health 263 (Medical *aid verification and claims)*	27 (55)
None	11 (23)
Dispense ware (*pharmacy dispensing)*	6 (12)
Medi-Plus (*medical billing and hospital management)*	2 (4)
ATA Finix (*payment processing and financial services)*	1 (2)
Consult (*manage patient consultations and clinical data)*	1 (2)
TriMed (*patient management and billing)*	1 (2)

### Resource availability, skill and knowledge readiness

Of the 49 health facilities that completed the online survey, only 5 (10%) had a designated HIO, and only 3 (6%) had staff who received formal training on the MoHCC HIS. Only 7 (14%) had a budget for training staff on HIS, and 20 (41%) were aware of the MoHCC HIS.

### Perceptions of healthcare providers towards MoHCC HIS reporting, 2024

Of the 49 health facilities, 21 (43%) reported that their staff believed reporting to MoHCC HIS would improve the quality of care, while 11 (22%) did not, and the remaining 17 (35%) were unsure. When asked whether the facilities’ staff believed that reporting would improve patient outcomes, 25 (51%) agreed that it would, 20 (41%) were neutral, and 4 (8%) believed it would not.

### Leadership readiness

Some facilities reported a commitment from leadership as a facilitator; one of the participants, a nurse, had this to say:

“Our leadership is very committed, as they are the ones who released us from work to attend the MoHCC HIS sensitisation workshop. Our leadership also provides us with the internet, power backup, and photocopies of the reporting tools for us” (Nurse 7). However, many respondents noted leadership weaknesses that hindered effective reporting to the MoHCC HIS. The quantitative data indicated that only 5 of 49 facilities (10%) had a designated Health Information Officer, and only 7 facilities (14%) allocated a budget for HIS training, illustrating a lack of prioritisation of reporting at the leadership level.

One administrator noted, “We need a dedicated Health Information Officer, but our financial limitations stop us from creating new positions. We need the personnel to ‘yacho yebasa’ (we need the staff to report through MoHCC HIS)” (Administrator 1). Likewise, nurse managers voiced their dissatisfaction regarding the absence of structured support: “The private sector is frequently neglected during HIS training sessions or workshops, and when fresh guidelines are issued… we are left to gather some of this new information independently” (Nurse Manager 7).

Leadership deficiencies were also evident in technical readiness. ICT personnel highlighted that external vendors governed private systems, restricting interoperability: “Tri Four decides how TriMed can engage with other applications. As a result, we are uncertain if TriMed will function in conjunction with MoHCC HIS” (Administrator 1).

### Facilitators for the successful integration of Zimbabwe’s private healthcare data into the MoHCC HIS, 2024 (Table 4)

The most frequently cited facilitators for integrating private health care data into MoHCC HIS were establishing public-private partnerships 25 (54%), availing government grants 9 (21%), and policy incentives 8 (19%). Some facilitators mentioned by a few facilities include having a government-employed HIO to collect the data 1 (2.5%).

**Table 4:** Barriers and facilitators for the successful integration of Zimbabwe’s private healthcare data into the MoHCC HIS, 2024.

• Facilitators	• Training of private healthcare professionals in the MoHCC HIS • Inclusion of private healthcare professionals in MoHCC HIS workshops • Interoperability of the private sector HIS and MoHCC HIS • Availing of tablets for MoHCC HIS reporting • Availing of tailor-made private sector HIS reporting tools such as the T5 • Availing of a MOHCC Health Information Officer (HIO) • Availability of Information, Communication and Technology (ICT) specialists in private healthcare facilities • Availability of TelOne and liquid Internet in private healthcare facilities • Availability of solar and diesel generators as a power backup and computers in private health care facilities
Barriers	• Incompatibility of private healthcare facilities’ HIS with MoHCC HIS • Time wasting due to zero reporting for services not offered at a facility • Lack of a designated health information officer • Lack of formal training in the MoHCC HIS • No participation in MoHCC HIS training • Concerns regarding patient data security • Medical staff are not transparent about their patients to the nursing staff who compile the statistics

From the qualitative data, private-sector key informants identified multiple factors that could improve their readiness to report to the MoHCC HIS. One significant concern raised by respondents was the lack of formal training for both paper-based and electronic MoHCC HIS systems.

One respondent stated, “It *is essential for us to receive formal training in both the paper and electronic formats of MoHCC HIS and to take part in the MoHCC HIS workshops so we can understand the tools and the deadlines for data submission. Furthermore, we require MoHCC officials to provide support and supervision at our sites to help overcome any challenges we may face.”* (Nurse Manager, 9)

Participants emphasised the necessity of seamless integration between private-sector EHRs and the MoHCC HIS. However, they recognised potential technical challenges as highlighted by one participant:

*“Our electronic system needs to be compatible with the MoHCC HIS to facilitate the easy sharing and retrieval of data. However, we may encounter integration difficulties since Tri Four restricts the interoperability of TriMed.” (ICT Person 2)*

Respondents raised concerns about the practicality of the MoHCC HIS reporting tools. They pointed out that some reporting requirements are not well-suited for private health facilities, leading to inefficiencies*:*

*“To promote ongoing reporting, the MoHCC HIS reporting tools should be specifically designed for private health facilities and eliminate unnecessary time expenditures. The zero reporting that we are required to perform for services on the Tally 5 that we do not provide is excessively time-consuming.” (Nurse Manager 5)*

A significant issue highlighted was the burden on healthcare personnel, which hindered their ability to dedicate time to data entry and reporting.

*“The MoHCC should assign a Health Information Officer (HIO) to our facility for reporting, as many of our nurses are overloaded with patient care. We also require a designated desktop computer for MoHCC HIS reporting to ensure that reporting does not disrupt the electronic systems we currently utilise.”* (Administrator 2)

Despite these obstacles, some facilities mentioned having the necessary resources to facilitate electronic reporting, including ICT professionals, reliable internet access, and backup power solutions. They preferred electronic reporting rather than traditional paper-based methods: *One* participant had this to say: *“We favour electronic reporting systems compared to paper-based alternatives.” (Nurse 3)*

Lastly, participants stressed the importance of governmental support, particularly in providing essential healthcare resources, as expressed by one participant: *“The government must provide resources to the private sector, including vaccines and free medications for treatment and free HIS training.” (Nurse 4)*

### Barriers to the successful integration of Zimbabwe’s private healthcare data into the MoHCC HIS (Table 4)

The most stated barriers to integrating private health care data into MoHCC HIS were insufficient government support 23 (47%), lack of incentives 14 (29%), and regulatory compliance issues 7 (14%). Other barriers indicated by facility 1 (2%) include a lack of government engagement and the need for additional staff to manage health information.

The private-sector key informants from the in-depth interviews voiced several concerns about their involvement in HIS training, data protection, system interoperability, reporting channels, and the availability of dedicated personnel for HIS reporting. Their voices reveal gaps that must be addressed to enhance their engagement in MoHCC HIS processes.

Numerous respondents felt marginalised from MoHCC HIS training sessions and workshops, especially when new guidelines were rolled out. This exclusion forced them to depend on self-study, which they regarded as insufficient.

One respondent noted, “The *private sector is consistently overlooked during HIS training sessions or workshops, and when new guidelines are released, such as those for antiretroviral therapy. We are left to gather some of the new information independently. We are only remembered in December when MoHCC prepares to publish statistics on babies born during Christmas.” (Nurse Manager 7)*

Another significant challenge was the protection and confidentiality of data, notably due to private doctors’ hesitance to share patient details with the nurses tasked with data collection. This created obstacles in reporting accurate figures, especially concerning HIV-related cases, as illustrated by the following statement: *“Private doctors are not inclined to share details about their patients with the nursing staff responsible for compiling statistics, which makes it challenging to gather data for reporting—such as statistics on HIV exposure in infants.” (Nurse 6)*

Several respondents highlighted their worries about privacy, underscoring the private sector’s reluctance to disclose sensitive patient information, as highlighted by one participant: *“We maintain privacy in our practice; therefore, we are apprehensive about the security of our patients’ data.” (Nurse 3)*

The integration of private-sector EHRs with the MoHCC HIS remains questionable. Participants noted that external vendors, such as Tri Four, control private systems like TriMed, thereby restricting their ability to achieve interoperability*. One participant had this to say: “Tri Four determines how TriMed can interact with other applications. Therefore, we are unsure if TriMed will be compatible with MoHCC HIS.” (Administrator 1)*

Participants also highlighted the ambiguity regarding the submission process for data, especially when using paper-based reporting systems: *“At present, there are no defined channels for us to submit the data, particularly with the paper-based system.” (Nurse Manager 4)*

Additionally, respondents stressed the importance of having dedicated HIOs to handle reporting duties. However, private facilities faced difficulties in establishing such roles due to budget limitations: *“We need to have a dedicated Health Information Officer, but our financial constraints prevent us from adding new positions. We require staff to ‘yacho yebasa’ (we need the staff to report through the MoHCC HIS). (Administrator 1)*

## Discussion

This assessment examined the organisational readiness of Zimbabwe’s private healthcare sector to report through the MoHCC HIS, as well as the key factors that facilitate or hinder the successful integration of private healthcare data into the system.

This assessment reveals a partial level of technological and institutional readiness among private health facilities in Zimbabwe for integration into the national HIS. This is evidenced by the availability of technological infrastructure in most facilities and the perceived benefits of reporting, such as improved quality of care and patient outcomes. Key facilitators identified by study respondents for improved reporting include strong public-private partnerships and customised reporting tools to minimise time wasted due to zero reporting. Perceived barriers were insufficient government support, as evidenced by a lack of training on the MoHCC HIS and/or current treatment guidelines for various health conditions. The findings highlight a critical gap between the existing reporting mechanisms and the vision for a comprehensive, data-driven health system [11]. To bridge this gap, it is essential to frame the path forward within the World Health Organisation’s Global Strategy on Digital Health 2020–2025, which provides a key normative guideline for digital health transformation worldwide [12].

The partial technological readiness in reporting was evidenced by facilities’ willingness to report, the availability of infrastructure, and the presence of some data management systems. Platforms owned by vendors had little integration, lowering technological readiness for change. The availability of technological equipment is critical to adopting the MoHCC HIS, as it helps ensure consistent reporting. This finding contrasts with findings from a study in Nigeria, which found that hospitals had no good infrastructure or computers and related technologies, and that the health workforce did not have computer-related skills to enable them to utilise electronic medical records (EMR) [13].

The private sector contributes significantly to the provision of health products and services, yet this group falls short in routine reporting. The WHO Global Health Strategy’s vision is to improve health for everyone by accelerating the development and adoption of appropriate, accessible, affordable, scalable and sustainable person-centric digital health solutions [12].

The current exclusion of private sector data directly impedes this vision in Zimbabwe, creating an incomplete picture of the national disease burden and hindering the ability to promote health and well-being through data [14]. Achieving better health service provision and better resource management is the ultimate goal of this readiness assessment, and it requires that all data sources be harnessed within an interoperable digital health ecosystem, a core concept outlined in the WHO Global Health Strategy. MoHCC does not support direct electronic reporting by private providers, as the system requires that facilities submit paper-based reports to the district officer, who then manually enters the data into DHIS2 [15]. However, private players reported being ready to enter data directly into DHIS2 as it saves time and resources compared to paper-based forms. Studies done elsewhere suggest ways to remedy this situation. Nigeria piloted a DHIS2 mobile project, intending to improve the mobile DHIS2 as a scalable platform that would allow the HIS to include data from the private sector [16]. Most private players in Nigeria face challenges with the availability of reporting tools and infrastructure, and when available, reporting rates and timelines from the private sector significantly improved [16].

The challenges identified, such as fragmented vendor platforms with limited integration, align with the WHO digital health strategy, which states that ill-coordinated or disjointed digital health initiatives lead to information fragmentation and, consequently, poor service delivery [12]. To counter this, the strategy emphasises that successful initiatives require an integrated strategy with strong governance and leadership. For Zimbabwe, this means the MoHCC’s digital health plan must explicitly include a governance framework for private-sector integration, moving beyond ad hoc reporting to a systematic, standards-based approach.

A robust commitment to ethical practices and security must accompany the expansion of data collection. The WHO digital health strategy clearly states that health data are classified as sensitive personal data that necessitate high safety and security standards and a regulatory framework to safeguard privacy, confidentiality, integrity, and availability [12]. Consequently, any integration plan must focus on the needs of individuals, foster trust, and ensure patient consent, as emphasised in the strategy. This underscores Zimbabwe’s dual responsibility to utilise data for the public good while vigorously protecting individual rights.

Private healthcare providers were found not to have paper-based reporting tools, which contributed to non-reporting [17]. As in our findings, the unavailability of health information personnel or managers, as well as computers and infrastructure in some facilities, was evident in most developing countries struggling with integrating private healthcare data into NHIS. The lack of established HIS positions and reliance on paper-based systems implies inadequate structural readiness. Facilities lack entrenched systems to support routine reporting, jeopardising sustainability [18].

Barriers to the successful integration of private healthcare data into the MoHCC HIS included unfavourable attitudes among nurses and doctors towards reporting, as several respondents perceived reporting as an unimportant component of service delivery [19]. Skill readiness among private health facilities in Zimbabwe was partial. Staff willingness to report existed, but limited training and knowledge hindered effective reporting. The variable skill readiness and need for tailored tools align with the strategy’s focus on digital health literacy and a capable health workforce [20]. The WHO strategy calls for placing people at the centre, which includes not only patients but also the health workers who must use these systems [20]. Investments in training and the development of modular, context-appropriate reporting tools (as piloted in Senegal) are concrete actions that directly support the WHO Global Health Strategy’s aim to advocate for people-centred health systems enabled by digital health [20]. This finding was also reported in South Africa, where a lack of staff interest in using computers indicated an unfavourable attitude towards HIS [21], and in Serbia, where doctors did not believe that using HIS would help them with their work [22]. Inadequate computer skills and perceived ease of use were contributors to unfavourable attitudes towards reporting in Saudi Arabia [19]. Unlike in other settings, private health practitioners in Zimbabwe had positive attitudes towards reporting, as evidenced by facilities that were already reporting immunisation and notifiable diseases through government health facilities in their catchment areas.

Some practitioners/facilities felt that reporting tools should be tailored to suit facilities that offer specialised care, so they are not required to spend time reporting on services they do not provide. Senegal implemented modular monthly reporting tools, with facilities that offered only family planning services receiving reporting forms that included only related indicators. At the same time, facilities were given credentials to DHIS2, so they only enter data related to the services offered [15].

Other barriers to NHIS reporting can include the generally low uptake of digital health packages in developing countries, both in public and private facilities [19]. However, in Harare, private health facilities were already using digital health platforms, such as Health 263 and TriMed, among others, suggesting a higher uptake.

Some key organisational factors, such as managerial support, technological preparedness, and employee awareness, were identified as potentially hindering the adoption of electronic health systems, and these factors can impact private-sector reporting to the MoHCC HIS [23]. Another study found that general barriers towards adoption of information systems were personnel, organisational, technological, and legal factors [24]. On the contrary, leaders/managers demonstrated commitment to reporting by using reporting tools and sending their staff to the recent HIS sensitisation meeting on the importance of reporting, held in Harare Province.

Furthermore, the private sector’s reported willingness to engage points to a crucial opportunity for public-private partnerships. The WHO strategy actively encourages collaboration, stating that maximising impact requires leveraging both new and existing collaborations and partnerships in the wider digital health ecosystem [12]. Formalising these partnerships, as suggested by our findings and the WHO strategy, is not just operational but strategic, aligning resources to ensure the sustainability and growth of digital health [12].

Another reported facilitator to private health facility reporting was the availability of tailor-made reporting tools to minimise time wasted due to zero reporting.,

This assessment offers several notable strengths that enhance its contribution to understanding private sector readiness for health information system integration in Zimbabwe. The mixed-methods approach combining qualitative insights with quantitative data provided a nuanced evaluation of reporting challenges and opportunities. These methodological choices strengthened the assessment’s relevance to policy planning and its potential to inform interventions to improve private sector reporting to the MoHCC HIS at scale.

However, the study’s findings must be interpreted in light of specific constraints. The sample size was limited by budgetary constraints, limiting the depth and diversity of insights, particularly from smaller or rural facilities that may face distinct challenges. Additionally, the online survey encountered a low response rate despite weekly reminders, potentially skewing results toward facilities with existing technological capacity. While the inclusion of facilities from multiple cities provided geographic diversity, expanding the sample to additional regions could have revealed broader disparities in readiness levels. These limitations underscore the need for future assessments to incorporate larger, more representative samples and hybrid data collection methods to improve response rates and generalisability.

## Conclusion

Private health facilities in Zimbabwe are partially ready to report to the MoHCC HIS. This is evidenced by their willingness to report and by the availability of essential infrastructure, such as computers, the internet, and some health information management systems. A barrier identified was insufficient or no government support for private health facilities to train on the MoHCC HIS.

Ongoing training programmes must be prioritised to ensure private facility staff are proficient in the MoHCC HIS tools and reporting timelines. Continuous engagement and new statutory instruments are needed to mandate comprehensive reporting from private providers. Public-private partnerships are critical to facilitate resource sharing, technical support, and collaborative problem-solving, complemented by regular supervision visits to address operational challenges. Interoperability between private sector platforms and the MoHCC HIS is essential to enable seamless data exchange.
