# Supplementary material for: An exploratory mixed-methods assessment of private health sector readiness for reporting through the Ministry of Health and Child Care Health Information System in Zimbabwe
Source: PLOS Digit Health. 2026 Jul 21;5(7):e0001566. doi: 10.1371/journal.pdig.0001566 (PMC13387566; doi:10.1371/journal.pdig.0001566)
Supplement: Attachment — Submitted filename: Response to Reviewers.docx [file pdig.0001566.s003.docx]

**Manuscript: [PDIG-D-25-00549R1] - [EMID:5d9b43868255c4ce]**

**Response to Reviewers'**

Dear Reviewers

Thank you very much for taking the time and effort to review our manuscript. We sincerely appreciate your thoughtful comments and valuable recommendations. We have carefully addressed all the comments, and our responses are provided below:

**Reviewer 3:**

|  | **Reviewer’s comments** | **Response** | **Page and line number** |
| --- | --- | --- | --- |
| 1 | Line 165-172, this list is confusing since this section should be about your Research questions, aims or objectives.  It should not be a list of things that you did, but it must be a list of what you aim to answer. | Thank you. We have corrected the list to show what we aimed to answer, and it now reads as:   1. Identify private facilities’ structural and technological readiness for reporting through the MoHCC HIS, 2024 2. Determine the resources, staff skills and knowledge readiness for reporting through the MoHCC HIS, 2024 3. Evaluate healthcare providers' perceptions towards integrating with the MoHCC HIS, 2024. 4. Determine leadership readiness for reporting through the MoHCC HIS, 2024 5. Identify key facilitators and barriers for successful integration of the private sector into the MoHCC HIS, 2024. | **Introduction**  Page 10  Line #166 -179 |
| 2 | Moreover, the wording of the heading in the results section does not align with the listed objectives.  For now, it is really difficult to read this paper because the listed objectives are unorganised and are not clearly mapped to the ORC framework. | Thank you. We have aligned the objectives and the results section. | **Introduction**  Page 10  Line #166 -179 |
| 3 | There needs to be a discussion of why the ORC framework is applicable to this study and how it leads to the following questions. | We have included a discussion on why the ORC framework is applicable to our study and how it led to the questions. Now reads as:  This paper provides a critical, evidence-based assessment to address this knowledge gap. Using a mixed-methods approach guided by the Organisational Readiness for Change framework, put forward by Holt (9). The study was guided by the Organisational Readiness for Change (ORC) framework [8,9], which conceptualises readiness as both the collective determination to implement change (change commitment) and shared confidence in the ability to do so (change efficacy). This framework was selected because it encompasses both psychological and structural dimensions of organisational change—essential for assessing private sector readiness for HIS integration, which requires not only technical infrastructure but also provider willingness to adopt new reporting practices.  The ORC framework directly shaped each research objective through its core constructs. Structural readiness informed Objective 1 on the facilities' infrastructure for reporting. Resource availability shaped Objective 2 on staff skills and resources. Psychological readiness underpinned Objective 3, which focused on provider perceptions of integration. Leadership readiness guided Objective 4 on leadership's role. The framework's emphasis on identifying facilitators and barriers directly shaped Objective 5. These constructs were operationalised in both quantitative and qualitative tools to ensure theoretical consistency | **Introduction**  Page 9  Line #  162-177 |
