# Supplementary material for: An exploratory mixed-methods assessment of private health sector readiness for reporting through the Ministry of Health and Child Care Health Information System in Zimbabwe
Source: PLOS Digit Health. 2026 Jul 21;5(7):e0001566. doi: 10.1371/journal.pdig.0001566 (PMC13387566; doi:10.1371/journal.pdig.0001566)
Supplement: Attachment — Submitted filename: Response to Reviewers-PLOS DIGITAL HEALTH.docx [file pdig.0001566.s002.docx]

**Response to Reviewers'**

Dear Reviewers,

Thank you very much for taking the time and making the effort to reviewing our manuscript. We appreciate the thoughtful comments and recommendations. We have addressed the comments as shown below. We hope the updated paper meets your standards and is now ready to be published in your distinguished journal.

Yours sincerely,

Memory Chimsimbe

**Reviewer 1:**

|  | **Reviewer’s comments** | **Response** | **Page and line number** |
| --- | --- | --- | --- |
| 1 | Does this manuscript meet PLOS Digital Health’s publication criteria? Is the manuscript technically sound, and do the data support the conclusions? The manuscript must describe methodologically and ethically rigorous research with conclusions that are appropriately drawn based on the data presented. Yes | Thank you |  |
| 2 | Has the statistical analysis been performed appropriately and rigorously? Yes | Thank you |  |
| 3 | Is the manuscript presented in an intelligible fashion and written in standard English? | Thank you |  |
| 4 | . Have the authors made all data underlying the findings in their manuscript fully available (please refer to the Data Availability Statement at the start of the manuscript PDF file)? Yes | Thank you |  |
| 5 | Methods section: Please inform the readers about the process of creating the quantitative questionnaire and the qualitative instruments. I suggest mentioning whether there were any validation processes, piloting, or testing of the questions in general, especially if they were extracted from other questionnaires and compiled. This will strengthen the data collection information and also showcase the creation and potential use of both instruments by others. | We have included details of the process of creating the quantitative questionnaire and the qualitative instruments. | **Data collection methods and tools**  29 —31  Line # 346-354 |
| 6 | Results: The results section follows a clear structure.  Do revise a few comments that should be moved to the discussion when the authors start "explaining" the reason for the findings.  Additionally, please consider a similar subtitle structure for the discussion | Thank you, we have removed the sentence explaining the reasons for the finding. | **Results**  14  Line #  506, 513-516 |
| 7 | Discussion: I suggest referring to the Global Digital Health Strategy by WHO as a key guideline for digital health worldwide, with special reference to data management, security, and the importance of public-private partnerships. The purpose of working on the country´s readiness is to achieve the ultimate goal of better health service provision. Better management? Yet also equally important to reflect on data protection and the good use of data from a people-centred approach. Reflecting on some sections of the strategy can further strengthen key elements presented by the authors. | We have referred to the WHO Global Digital Health Strategy as a key guideline for digital health worldwide, with particular emphasis on data management, security, and the importance of public-private partnerships. | **Discussion**  21-27  Line #  748-751,  776-784,  811-817 |

**Reviewer 2:**

|  | **Reviewer’s comments** | **Response** | **Subtopic and page number** |
| --- | --- | --- | --- |
| 1 | Does this manuscript meet PLOS Digital Health’s publication criteria? Is the manuscript technically sound, and do the data support the conclusions? The manuscript must describe methodologically and ethically rigorous research, with conclusions that are appropriately drawn from the data presented.  Partly | Thank you. We have revised the manuscript in response to the reviewers' comments. |  |
| 2 | Has the statistical analysis been performed appropriately and rigorously? Yes | Thank you |  |
| 3 | Is the manuscript presented in an intelligible fashion and written in standard English? | Thank you |  |
| 4 | Have the authors made all data underlying the findings in their manuscript fully available (please refer to the Data Availability Statement at the start of the manuscript PDF file)? Yes | Thank you |  |
| 5 | This paper has significant methodological and structural shortcomings that substantially limit its contribution to the field.  - Caption/Objective: Clarify the study's primary objective. The current caption fails to communicate the research purpose effectively. | We have rephrased the objective. | **Introduction**  9  Line # 174-190 |
| 6 | Reorganise by creating a separate section for healthcare system description (private/public structures and their differences) with enhanced detail. The current introduction introduces too many associated structures without adequate context, making comprehension difficult. Additionally, elaborate on existing system weaknesses and clearly articulate this paper's specific contribution. | We have reorganised by creating a separate section for the healthcare system description (private/public structures and their differences), with greater detail. | **Introduction**  5—10  Line # 90-160 |
| 7 | Examples of methodological shortcomings: - Justify the chosen mixed-methods study design | We have provided the justification | **Study design**  Page 14  Line # 277-282 |
| 8 | Detail participant recruitment procedures | We have detailed participant recruitment procedures. | **Materials and methods**  Pages 15-17  Line # 322-342 |
| 9 | Specify validated instruments used for data collection |  | **Data collection methods and tools**  Pages 17-19  Line # 344-392 |
| 10 | Explain data cleaning procedures, particularly for subjective interview data. | We have explained the data cleaning procedures, particularly for subjective interview data | **Data analysis**  Page 21-22  Line #437-439,  443-452 |
| 11 | Clarify the integration strategy for combining qualitative and quantitative data. | We have clarified the integration strategy for combining qualitative and quantitative data | **Data analysis**  Page 22  Line # 462-466 |
| 12 | The quantitative analysis relies solely on descriptive statistics without significance testing, preventing meaningful conclusions from being drawn | We thank the reviewer for this observation. The use of descriptive statistics without inferential testing was an intentional choice aligned with the **exploratory purpose** of this study. Our goal was not to test hypotheses but to map the landscape of private-sector readiness using a purposively sampled group (n=49). Significance testing would not be appropriate or meaningful given the non-random sampling strategy. Instead, we relied on **triangulation with rich qualitative data** to contextualize and explain the descriptive patterns, providing actionable insights for policy and planning. We acknowledge this limitation in the revised manuscript. | **Study design**  Page 14  Line #274-282,  **Discussion**  Page 42  Line 840-842 |
| 13 | The interpretation of interview data lacks rigour and transparency. The methods section must include transcription procedures, analytical framework employed, and systematic approaches to qualitative data analysis. | We have included the transcription procedures, analytical framework employed, and systematic approaches to qualitative data analysis | **Data analysis**  Page 22  Line #  443-452 |
| 14 | Current sections merely summarize descriptive statistics rather than provide analytical interpretation. Strengthen the discussion by contextualising findings within existing literature and drawing substantive conclusions from the data. | We have strengthened the discussion | **Discussion**  Page 36 —42  Line #696-842 |
| 15 | Additionally, the reference list exhibits issues with consistency, completeness, and accuracy. | We have corrected the reference list. | **Reference**  Pages 48 —50  Line # 998 - 1066 |
| 16 | While this paper addresses an important aspect of healthcare data sharing between private and public sectors, the substantial methodological flaws and analytical limitations render it unsuitable for publication in PLOS Digital Health in its current form. | Thank you for your feedback on our manuscript. We have carefully considered your concerns regarding methodological and analytical rigor, and we are fully committed to undertaking a comprehensive revision to address these points in line with the journal’s standards. |  |

**Reviewer 3:**

|  | **Reviewer’s comments** | **Response** | **Subtopic and page number** |
| --- | --- | --- | --- |
| 1 | Does this manuscript meet PLOS Digital Health’s publication criteria? Is the manuscript technically sound, and do the data support the conclusions? The manuscript must describe methodologically and ethically rigorous research with conclusions that are appropriately drawn based on the data presented.  Partly | Thank you. We have revised the manuscript in response to the reviewers' comments, as shown in the two comment templates. |  |
| 2 | Has the statistical analysis been performed appropriately and rigorously? Yes | Thank you |  |
| 3 | Is the manuscript presented in an intelligible fashion and written in standard English? | Thank you |  |
| 4 | Have the authors made all data underlying the findings in their manuscript fully available (please refer to the Data Availability Statement at the start of the manuscript PDF file)? Yes | Thank you |  |
| 5 | Title 1. Please revise the title to reflect the mixed-method approach of the study. | An exploratory mixed-methods assessment of private health sector readiness for reporting through the Ministry of Health and Child Care Health Information System in Zimbabwe | **Title page**  Page 1  Line # 2-4 |
| 6 | Abstract 2. Make the abstract a single paragraph. contribution. | We have made the abstract a single paragraph | **Abstract**  Page 3  Line # 39-67 |
| 7 | Examples of methodological shortcomings: - Provide justification for the chosen mixed-methods study design | This approach enabled a comprehensive assessment of readiness levels while providing detailed insights into the systemic, operational, and attitudinal elements that affect data integration. As a result, it delivered a strong, triangulated evidence foundation to guide practical policy and practice suggestions aimed at improving the private sector's involvement in national health reporting. | **Study design**  Page 14  Line # 274-282 |
| 8 | Introduction 3. Need citation for "5,377 registered health facilities." You need to specify that even it is the same as citation 2. | We have included a citation for the 5,377 registered facilities | **Introduction**  Page 6  Line # 121 |
| 9 | For paragraph 3, citations are needed. | We have included citations | **Introduction**  Page 7  Line # 118-133 |
| 10 | There needs to be explicit research questions or objectives in the paper. Please list them towards the end of the introduction. This will help guide the structure of the results section. | We have listed the objectives at the end of the introduction | **Introduction**  Page 9-10  Line # 175-182 |
| 11 | Results 6. The main headings can be used as a basis for your research questions/objectives | The main headings are the basis for our research objectives | **Results**  Pages 23-36  Line # 468-694 |
| 12 | Lines 216 and 253 mention the same results. Please consider rewriting it to reduce redundancy. | We have rewritten to reduce redundancy | **Results**  Page 13  Line # |
| 13 | For lines 264-270, is there no critical feedback? You only used 1 quote and I don't think that is sufficient to describe the theme that you are describing. |  | **The role of leadership in Zimbabwe's private healthcare facilities in reporting through the MoHCC HIS**  Pages 15-16  Line # 359-361,  Pages 29-30  571-595 |
| 14 | Please create a table that lists the barriers and facilitators. | We have included a table with the barriers and facilitators | **Table 4: Barriers and facilitators for the successful integration of Zimbabwe's private healthcare data into the MoHCC HIS, 2024**  Pages  31-32  Line # 601-602 |
| 15 | Please present the results based on the Organisational Readiness for Change framework. It is unclear to what extent this framework influenced the survey design and results. . | Thank you. Our results are written based on the organisation's readiness constructs | **Results**  Pages  26-36  Line # 504-694 |
| 16 | Materials and methods 10. Provide more details on the interview procedure. Was this in-person or online? | Thank you. We have included further details on the hybrid nature of the data collection process. | **Data collection methods and tools**  Pages 17-20  Line # 373-392,  Line #395-404 |
| 17 | Where are the tables and graphs that you mentioned in line 567? | We have deleted the word graphs. The tables referred to are Tables 1 to 4 in the results section. | **Results**  Pages  26-36  Line # 504-694 |
| 18 | The qualitative analysis needs more details. For instance, how did you develop your code? How many of the authors were involved in the analysis? How did you manage agreement/disagreement in your codes and themes? | We have included the detailed qualitative analysis section in our methods | **Data collection methods and tools**  **Qualitative component**  Pages 30—31  Line # 394-421 |
| 19 | Was there an attempt to synthesise the quantitative and qualitative data? If yes, please explain the process. | There was an attempt to synthesise the quantitative and qualitative data | **Data analysis**  Page 20-21  Line # 417-421  And  **Discussion** |
